# The Relationship between Body Mass Index and In-Hospital Mortality in Bacteremic Sepsis

**DOI:** 10.3390/jcm12113848

**Published:** 2023-06-04

**Authors:** Shalom Lebovitz, Guy Rozen, Zahi Abu Ghosh, Maya Korem, Hila Elinav, Hiba Zayyad, Shemy Carasso, David Planer, Offer Amir, Gabby Elbaz-Greener

**Affiliations:** 1 Department of Cardiology, Hadassah Medical Center, Jerusalem 9112001, Israel; 2 Faculty of Medicine, Hebrew University of Jerusalem, Jerusalem 9190401, Israel; 3 Cardiovascular Center, Tufts Medical Center, Boston, MA 02111, USA; 4 Cardiac Arrhythmia Center, Massachusetts General Hospital, Harvard Medical School, Boston, MA 02115, USA; 5 Department of Clinical Microbiology and Infectious Diseases, Hadassah Medical Center, Jerusalem 9112001, Israel; 6 Infectious Diseases Unit, The Baruch Padeh Medical Center Poriya, Tiberias 1528001, Israel; 7 The Jerusalem Integrated Heart Center, Shaare Zedek Medical Center, Jerusalem 9103102, Israel

**Keywords:** BMI, sepsis, bacteremia, bacteremic sepsis, BMI mortality, obesity paradox

## Abstract

Background: The association between Body Mass Index (BMI) and clinical outcomes following sepsis continues to be debated. We aimed to investigate the relationship between BMI and in-hospital clinical course and mortality in patients hospitalized with bacteremic sepsis using real-world data. Methods: A sampled cohort of patients hospitalized with bacteremic sepsis between October 2015 and December 2016 was identified in the National Inpatient Sample (NIS) database. In-hospital mortality and length of stay were defined as the relevant outcomes. Patients were divided into 6 BMI (kg/m^2^) subgroups; (1) underweight ≤ 19, (2) normal-weight 20–25, (3) over-weight 26–30, (4) obese I 31–35, (5) obese II 36–39, and (6) obese stage III ≥ 40. A multivariable logistic regression model was used to find predictors of mortality, and a linear regression model was used to find predictors of an extended length of stay (LOS). Results: An estimated total of 90,760 hospitalizations for bacteremic sepsis across the U.S. were analyzed. The data showed a reverse-J-shaped relationship between BMI and study population outcomes, with the underweight patients (BMI ≤ 19 kg/m^2^) suffering from higher mortality and longer LOS as did the normal-weight patients (BMI 20–25 kg/m^2^) when compared to the higher BMI groups. The seemingly protective effect of a higher BMI diminished in the highest BMI group (BMI ≥ 40 kg/m^2^). In the multivariable regression model, BMI subgroups of ≤19 kg/m^2^ and ≥40 kg/m^2^ were found to be independent predictors of mortality. Conclusions: A reverse-J-shaped relationship between BMI and mortality was documented, confirming the “obesity paradox” in the real-world setting in patients hospitalized for sepsis and bacteremia.

## 1. Introduction

Body mass index (BMI) is a commonly utilized index for characterizing weight status in clinical and research settings despite it not differentiating between adipose and muscular tissue [1]. Six BMI groups are currently defined by the World Health Organization (WHO); under-weight (BMI ≤ 18.5 kg/m^2^); normal-weight (BMI 18.5–24.9 kg/m^2^); over-weight (BMI 25–29.9 kg/m^2^); obese class I (BMI 30–34.9 kg/m^2^); obese class II (BMI 35–39.9 kg/m^2^); obese stage III (BMI ≥ 40 Kg/m^2^) [2,3]. Higher BMI was associated with an increased risk of overall mortality as well as many case-specific causes of death in a large study of the general population in the UK [3]. Higher BMI during adolescence has been associated with adult mortality from infectious diseases [4]. While these studies appraise the lifetime risk of mortality, they do not predict survival during acute events.

Multiple studies have investigated the effect of different BMI-grouped cohorts on short and long-term mortality rates in patients hospitalized with bacteremia, sepsis, and septic shock [5]. While some studies suggested that obesity independently portends negative outcomes [6,7], others showed that the higher-BMI groups might have a more favorable course [8,9]. The phenomenon of obesity having a protective effect from negative outcomes has been referred to as the ‘obesity paradox’. The existence of a true paradox and the theories explaining its occurrence have been highly debated.

In this study, we aimed to find predictors of negative outcomes in patients with bacteremic sepsis during the initial hospitalization and analyze the outcomes between the different BMI subgroups.

## 2. Materials and Methods

The data were drawn from the National Inpatient Sample (NIS), the Healthcare Cost and Utilization Project (HCUP), and Agency for Healthcare Research and Quality (AHRQ) [10]. The NIS database only includes de-identified data; therefore, this study was considered exempt from institutional review by the Human Research Committee. 

The NIS is the largest database of all-payer hospitalizations in the United States (U.S.) and represents an approximate 20% stratified sample of all inpatients admitted to U.S. hospitals [11]. This includes information at the hospital level, such as hospital region, teaching status, bed size, and cost of hospitalization, and other data at the patient level, including demographic characteristics, primary and secondary diagnoses and procedures, comorbidities, and length of stay (LOS). National estimates can be calculated using the patient-level and hospital-level sampling weights that are provided by the HCUP. 

Using data from the NIS, we created a database of relevant patients hospitalized between the years 2015 and 2016 for use in several studies examining outcomes in several disease states and BMI groups [12,13,14,15]. The data was extracted and analyzed with similar methods in all studies. The International Classification of Diseases, 10^th^ Revision, Clinical Modification (ICD-10-CM) was used from the last quarter of 2015 and thereafter for reporting diagnoses and procedures in the NIS database during the study period. For each index hospitalization, the database provides a principal discharge diagnosis and a maximum of 14 or 24 additional diagnoses, in addition to a maximum of 15 procedures. We restricted our cohort to the period during which data was coded with ICD-10 codes because the ICD-10 system includes individual codes for BMI values and ranges. 

We identified patients 18 years of age or older with a final diagnosis based on ICD-10 indicating bacteremic sepsis: 78.81, R65.20, R65.21, A40, A40.0, A40.01, A40.02, A40.3, A40.8, A40.9, A41.X (1–5), A41.50, A41.51, A41.52, A41.53, A41.58, A42.7, A22.7, B37.7, A26.7, A28.2, A54.86, B00.7, A32.7, A24.1, A39.2-A39.4, A20.7, A21.7, A48.3–as “I10_Dx1. The following codes represent the six BMI subgroups we have created for our study: Z68.1, BMI ≤ 19 kg/m^2^, under-weight group; Z68.20–25, BMI 20–25 kg/m^2^, normal-weight group; Z68.26–30, BMI 26–30 kg/m^2^, over-weight group; Z68.31–35, BMI 31–35 kg/m^2^, obese I group; Z68.36–39 kg/m^2^, BMI 36–39, obese II group; Z68.4, BMI ≥ 40 kg/m^2^, obese stage III group.

The following patient demographics were collected from the database: age, sex, and race. Prior comorbidities were identified by measures from the AHRQ. For calculating Deyo-Charlson Comorbidity Index (Deyo-CCI), additional comorbidities were identified from the database using ICD-10 cm codes. Deyo-CCI is a modification of the Charlson Comorbidity Index, containing 17 comorbid conditions of differential weights, with a total score ranging from 0 to 33 (detailed information on Deyo-CCI is provided in Appendix A Table A1). Higher Deyo-CCI scores indicate a greater burden of comorbid diseases and are associated with mortality 1 year after admission [16]. The index has been used extensively in studies from administrative databases, with proven validity in predicting short- and long-term outcomes [17,18]. Our primary outcome in this study was in-hospital mortality. Length of stay in the hospital was measured as a secondary outcome. 

The chi-square (χ2) and Wilcoxon rank sum tests were used to compare categorical variables and continuous variables, respectively. The NIS provides discharge sample weights that are calculated within each sampling level as the ratio of discharges in the universe to discharges in the sample [19]. 

We generated a weighted logistic regression model to identify independent predictors of in-hospital mortality. Candidate variables included patient-level characteristics, Deyo-CCI, and hospital-level factors. We included all candidate variables that were associated with our primary and secondary outcomes in our final multivariable regression model. A linear regression model was used to identify predictors of LOS. 

For all analyses, we used SAS^®^ software version 9.4 (SAS Institute Inc., Cary, NC, USA). A *p*-value < 0.05 was considered statistically significant.

## 3. Results

### 3.1. Study Cohort

A total sample of 18,152 hospitalizations for bacteremic sepsis across the U.S. during 2015 (last quarter) and 2016 were included in the analysis. After implementing the weighting method, these represented an estimated total of 90,760 hospitalizations for bacteremic sepsis during the index hospitalization. The majority of patients (57.0%) were female, and the mean age of the cohort was 64 ± 34.1 years. In this study, patients from all defined BMI categories were well represented.

### 3.2. Patient Characteristics

Demographic and clinical characteristics are shown in detail in Table 1. Female predominance and increased rates of comorbidities such as chronic obstructive pulmonary disease, chronic renal disease, atrial fibrillation/flutter, and higher Deyo-CCI scores were observed in both under-weight (BMI ≤ 19 kg/m^2^) and obese stage III groups (BMI ≥ 40 kg/m^2^) (Table 1). 

### 3.3. Length of Stay and Mortality Per BMI Groups 

**Figure 1:**
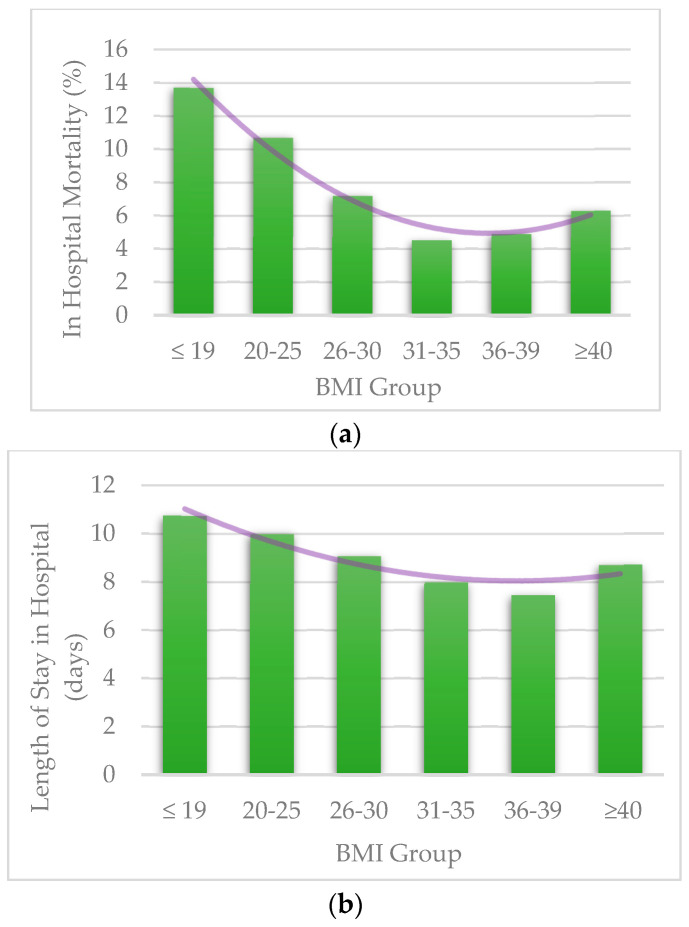
(**a**) Mortality per BMI groups; (**b**) Length of Stay per BMI groups. BMI = Body Mass Index.

A reverse-J relationship was found between the BMI and the study outcomes. Underweight patients (BMI ≤ 19 kg/m^2^) had higher mortality and longer LOS, as did the normal-weight patients (BMI 20–25 kg/m^2^) when compared to the higher BMI groups (Figure 1). The seemingly protective effect of a higher BMI diminished in the highest BMI group (BMI ≥ 40 kg/m^2^). The overall mortality rate during the study period was 7.7%, with a noticeably higher rate in under-weight (13.7%) and normal-weight (10.7%) patient population groups (*p* < 0.001) (Table 1). The overall mean LOS was 9.00 ± 0.07 days. Longer LOS was documented in the under-weight (10.74 ± 0.21 days), normal weight (9.99 ± 0.19 days), and over-weight subgroup (9.08 ± 0.19 days) (*p* < 0.001) (Table 1).

### 3.4. Predictors of In-Hospital Mortality

Univariate and multivariate analysis identified male gender, older age, increasing Deyo-CCI score, chronic renal failure, atrial fibrillation/flutter, and congestive heart failure as predictors of in-hospital mortality (*p* < 0.001) (Table 2). 

A reverse-J relationship between BMI and mortality was seen in the multivariable analysis. BMI ≤ 19 kg/m^2^ predicted in-hospital mortality after adjusting for potential confounders, with the highest risk of mortality occurring in underweight patients. (OR 1.35; [95% CI 1.25–1.47], *p* < 0.001). Excluding the underweight group, all other BMI ranges were associated with lower odds of mortality as compared to the index range of BMI 20–25 kg/m^2^ (*p* < 0.001) (Table 3).

### 3.5. Predictors of Length of Stay in Hospital

A reverse-J relationship between BMI and the secondary outcome of LOS was seen in univariable and multivariable analyses (Appendix A Table A2 and Table A3). In the multivariate analysis, we found that demographic characteristics such as age above 60, male gender, Deyo-CCI score of 2 or higher, atrial fibrillation/flutter, renal failure, and congestive heart failure were all associated with longer LOS (*p* < 0.001) (Appendix A Table A2).

In multivariable analysis after adjusting for potential confounders, BMI < 19 kg/m^2^ was associated with a longer LOS in the underweight patients compared to obese stage III patients (Mean LOS 9.73; [95% CI 9.34,10.12], *p* < 0.001 vs. Mean LOS 7.34; [95% CI 7.05,7.63], *p* < 0.001, respectively). Excluding the underweight group, all other BMI ranges were associated with shorter LOS as compared to the index range of BMI 20–25 kg/m^2^ (*p* < 0.001) (Appendix A Table A3).

## 4. Discussion

In this paper, using real-world data from a large U.S. database, we aim to better describe the controversial ‘obesity paradox’ for inpatients with a diagnosis of bacteremic sepsis. To the best of our knowledge, this is the largest study analyzing the relationship between BMI and mortality in patients hospitalized for bacteremic sepsis, including a weighted total of 90,760 hospitalizations. After adjusting for confounders, the underweight group (BMI ≤ 19 kg/m^2^) predicted in-hospital mortality and prolonged LOS with significantly better outcomes in the obese subgroups. Interestingly, the protective effect of a higher BMI diminished in the highest BMI group (BMI ≥ 40 kg/m^2^) in comparison to the normal weight group as well as the obese I and obese II groups yet remained a protective factor.

Multiple studies have investigated the effect of different BMI-grouped cohorts on short and long-term mortality rates in patients hospitalized with bacteremia, sepsis, and septic shock [5]. While some studies suggested that obesity independently portends negative outcomes [6,7], others showed that the higher-BMI groups might have a more favorable course [8,9]. This phenomenon has been referred to as the ‘obesity paradox’. One proposed explanation for this paradox is that patients with higher BMI have a blunted inflammatory cytokine response, possibly due to adipose tissue modulation of the immune response or a chronic inflammatory state [20]. Another theory proposes that the nutritional reserves of obese and overweight patients protect them from the adverse effects of catabolic processes during critical states, such as sepsis. One study suggests that initiating early enteral nutrition during critical disease confers near similar protection as in the higher BMI groups [21]. Another possible reason for the survival differences could be weight-based variations in medication and fluid dosing in these critically ill patients, as current sepsis guidelines recommend dosing according to weight, which can impact outcomes for BMI outliers [22]. 

While these may explain the protective effects of higher BMI, it seems that the underweight have increased mortality and morbidity. A growing body of research attributes these negative outcomes to the deleterious effects of sarcopenia and skeletal muscle wasting [23]. This has been studied in hospitalized patients, usually based on US or CT imaging of muscle mass in multiple populations as well as in sepsis, and clearly trends towards negative outcomes in muscle-depleted patients [23,24]. Several studies indicate that these measures may better predict outcomes when compared to BMI alone in specific populations, e.g., cancer patients [25] and elderly patients in ICU settings [26], yet it remains to be seen whether these predictors hold up in more heterogenous populations, age groups, and disease states. 

Among this study’s strengths are the use of real-world national data from a large heterogenic population with well-represented groups of obese stage III and underweight patients, which were under-represented in previously conducted studies. 

In order to fully represent all patient BMI groups, our study specifically included an underweight, a normal weight, and an obese stage III group, contrary to previous studies. Our results showed that underweight patients (BMI ≤ 19 kg/m^2^) have the highest risk of mortality compared to the other BMI groups, and we believe that this knowledge should be considered in mortality risk scores.

This study population represents the entire nationwide population of patients who were hospitalized with bacteremic sepsis in the US between October 2015 and December 2016, hence eliminating the selection bias in some of the prior studies regarding this population. 

To eliminate selection bias, the patient population in this study consisted of a large nationwide cohort of patients hospitalized for bacteremic sepsis in the U.S. during a set time period.

Several limitations should be considered when interpreting this study. First, the NIS database is a retrospective administrative database that contains patient records at the time of discharge, and coding errors may occur. Omitted from the collected data are multiple clinical variables, including vital signs, blood glucose levels, prognostication scores such as the SOFA score, medications, blood tests such as levels of CRP, ESR, and other markers of inflammation, all of which have been independently linked with adverse events, therefore we cannot rule out residual confounding of the associations we observed. Other important data absent from the NIS database includes specific treatment provided in hospitals and geographic variations of these practices. While this study sheds light on the relationship between BMI and sepsis with proven bacteremia, it does not detail the cultured organisms, sepsis severity scoring systems, or infection sources. Furthermore, it is likely that cases of secondary sepsis occurring later during an unrelated hospitalization have been included in the study, and therefore, results do not reflect primary cases of sepsis alone. 

Finally, the data collected does not include mortality past 30 days and other outcomes and only reflects the course of the initial hospitalization. The limitations noted above are balanced by the broad real-world data we utilized, which eliminates center-specific bias.

In conclusion, a reverse J-shaped relationship between BMI and mortality was seen in patients hospitalized for bacteremic sepsis in this study. Due to this, it may be warranted to consider BMI as part of the risk assessment for patients admitted with bacteremic sepsis.

## Figures and Tables

**Table 1:** Frequency distribution of demographic characteristics and outcomes of patients with bacteremic sepsis by BMI group.

BMI, *n*	≤19	20–25	26–30	31–35	36–39	>40	Total	*p*-Value
Unweighted	2888	2234	1852	2519	1890	6769	18,152	
Weighted	14,440	11,170	9260	12,595	9450	33,845	90,760	
**Age Group, %**								<0.001
18–44 yrs	10.9	9.2	7.9	10.6	11.0	13.3	11.3	
45–59 yrs	19.1	19.2	21.2	21.1	25.0	30.6	24.5	
60–74 yrs	29.5	31.0	38.0	40.8	42.4	42.3	38.2	
75 yrs or older	40.5	40.5	32.9	27.4	21.6	13.8	26.0	
**Gender, %**								<0.001
Female	52.4	44.7	53.5	55.1	58.4	64.4	57.0	
Male	47.6	55.1	46.4	44.9	41.5	35.5	42.9	
**Race, %**								<0.001
White	61.1	62.7	62.7	64.2	66.3	69.4	65.5	
Non-white	33.1	30.4	31.2	29.1	26.9	25.0	28.4	
**Comorbidity, %**								
Hypertension	31.5	35.8	42.2	45.3	48.0	43.4	41.2	<0.001
Congestive Heart Failure	12.9	12.0	13.4	15.4	17.7	21.7	17.0	<0.001
Diabetes Mellitus	14.8	20.1	30.8	39.1	42.4	44.9	34.6	<0.001
Renal Failure	22.5	24.9	29.9	31.3	29.6	32.4	29.2	<0.001
Chronic Pulmonary Disease	30.5	19.4	23.7	25.2	29.5	32.4	28.3	<0.001
Peripheral Vascular Disorders	9.7	9.8	10.5	10.5	10.1	8.5	9.5	<0.001
Atrial Fibrillation/Flutter	20.6	22.1	21.6	23.0	23.1	24.8	23.0	<0.001
Prior MI	3.8	4.7	5.3	7.1	5.8	4.7	5.1	<0.001
VT/VF	2.4	1.8	1.9	2.0	1.6	2.3	2.1	<0.001
**Deyo-CCI, %**								<0.001
0	12.7	12.7	13.6	13.4	13.1	12.1	12.7	
1	15.9	15.2	15.7	18.4	18.5	16.6	16.7	
2 or higher	71.4	72.2	70.7	68.2	68.5	71.3	70.6	
**Mortality, %**	13.7	10.7	7.2	4.5	4.9	6.3	7.7	<0.0001
**Length of Stay (days), Mean + SEM**	10.74 ± 0.21	9.99 ± 0.19	9.08 ± 0.19	7.99 ± 0.16	7.45 ± 0.16	8.71 ± 0.11	9.00 ± 0.07	<0.0001

BMI = Body Mass Index; MI = Myocardial Infarction; VT = Ventricular Tachycardia; VF = Ventricular Fibrillation; Deyo-CCI = Deyo Comorbidity Index; SEM = Standard Error of the Mean.

**Table 2:** Predictors of In-Hospital mortality for bacteremic sepsis (univariate).

Predictors	Odds Ratio (95% CI)	*p*-Value
**Age Group, yrs**		<0.001
18–44 yrs	1.00 (reference)	N/A
45–59 yrs	1.31 (1.18,1.46)	<0.001
60–74 yrs	1.73 (1.57,1.91)	<0.001
75 yrs or older	2.28 (2.06,2.52)	<0.001
**BMI Group**		<0.001
Below 20	1.33 (1.23,1.43)	<0.001
20–25	1.00 (reference)	N/A
26–30	0.65 (0.58,0.71)	<0.001
31–35	0.39 (0.35,0.43)	<0.001
36–39	0.43 (0.39,0.48)	<0.001
40 and above	0.56 (0.52,0.61)	<0.001
**Deyo-CCI**		<0.001
1	1.00 (reference)	N/A
0	0.83 (0.73,0.93)	0.001
2 or higher	1.85 (1.72,2.00)	<0.001
**Gender**		<0.001
Male	1.00 (reference)	N/A
Female	0.78 (0.75,0.82)	<0.001
**Race**		<0.001
Non-white	1.00 (reference)	N/A
White	0.85 (0.80,0.89)	<0.001
***Comorbidities***		
**Atrial Fibrillation/Flutter**		<0.001
No	1.00 (reference)	N/A
Yes	1.69 (1.61,1.79)	<0.001
**Chronic pulmonary disease**		<0.001
No	1.00 (reference)	N/A
Yes	0.90 (0.85,0.95)	<0.001
**Congestive heart failure**		<0.001
No	1.00 (reference)	N/A
Yes	1.28 (1.20,1.36)	<0.001
**Diabetes Mellitus**		<0.001
No	1.00 (reference)	N/A
Yes	0.66 (0.63,0.70)	<0.001
**Hypertension**		<0.001
No	1.00 (reference)	N/A
Yes	0.58 (0.55,0.62)	<0.001
**Peripheral vascular disorders**		0.697
No	1.00 (reference)	N/A
Yes	1.02 (0.94,1.10)	0.697
**Renal failure**		<0.001
No	1.00 (reference)	N/A
Yes	1.36 (1.29,1.43)	<0.001

CI = Confidence Interval; BMI = Body Mass Index; Deyo-CCI = Deyo Comorbidity Index; N/A = Not applicable.

**Table 3:** Predictors of In-Hospital mortality for bacteremic sepsis (multivariate).

Predictors	Odds Ratio (95% CI)	*p*-Value
**Age Group, yrs**		<0.001
18–44 yrs	1.00 (reference)	N/A
45–59 yrs	1.27 (1.13,1.43)	<0.001
60–74 yrs	1.72 (1.54,1.92)	<0.001
75 yrs or older	1.91 (1.70,2.14)	<0.001
**BMI Group**		<0.001
Below 20	1.35 (1.25,1.47)	<0.001
20–25	1.00 (reference)	N/A
26–30	0.66 (0.59,0.73)	<0.001
31–35	0.42 (0.38,0.47)	<0.001
36–39	0.48 (0.43,0.54)	<0.001
40 and above	0.66 (0.61,0.72)	<0.001
**Gender**		<0.001
Male	1.00 (reference)	N/A
Female	0.84 (0.80,0.89)	<0.001
**Race**		0.004
Non-white	1.00 (reference)	N/A
White	0.92 (0.87,0.97)	0.004
**Deyo-CCI**		<0.001
1	1.00 (reference)	N/A
0	0.91 (0.81,1.03)	0.149
2 or higher	1.74 (1.60,1.89)	<0.001
***Comorbidities***		
**Atrial Fibrillation/Flutter**		<0.001
No	1.00 (reference)	N/A
Yes	1.58 (1.49,1.67)	<0.001
**Congestive heart failure**		0.006
No	1.00 (reference)	N/A
Yes	1.10 (1.03,1.17)	0.006
**Chronic pulmonary disease**		<0.001
No	1.00 (reference)	N/A
Yes	0.82 (0.77,0.87)	<0.001
**Diabetes Mellitus**		**<0.001**
No	1.00 (reference)	N/A
Yes	0.66 (0.62,0.70)	<0.001
**Hypertension**		**<0.001**
No	1.00 (reference)	N/A
Yes	0.69 (0.65,0.73)	<0.001
**Peripheral vascular disease**		**<0.001**
No	1.00 (reference)	N/A
Yes	0.80 (0.73,0.87)	<0.001
**Renal failure**		**<0.001**
No	1.00 (reference)	N/A
Yes	1.15 (1.08,1.22)	<0.001

CI = Confidence Interval; BMI = Body Mass Index; Deyo-CCI = Deyo Comorbidity Index; N/A = Not applicable.

## Data Availability

The Healthcare Cost and Utilization Project (HCUP) Data Use Agreement does not allow us to make the data from the NIS database used for this study available to others. The NIS database is available for purchase by the public, and our detailed and transparent description of methods used for the data analysis allows anyone who wishes to do so to reproduce our results.

## Appendix A

**Table A1:** Deyo comorbidity index.

ICD-10 CM Codes	Condition	Score
I21.x, I22.x, I25.2	Myocardial infarction	1
I09.9, I11.0, I13.0, I13.2, I25.5, I42.0, I42.5-I42.9, I43.x, I50.x, P29.0	Congestive heart failure	1
I70.x, I71.x, I73.1, I73.8, I73.9, I77.1, I79.0, I79.2, K55.1, K55.8, K55.9, Z95.8, Z95.9	Peripheral vascular disease	1
G45.x, G46.x, H34.0, I60.x-I69.x	Cerebrovascular disease	1
F00.x-F03.x, F05.1, G30.x, G31.1	Dementia	1
I27.8, I27.9, J40.x-J47.x, J60.x-J67.x, J68.4, J70.1, J70.3	Chronic pulmonary disease	1
M05.x, M06.x, M31.5, M32.x-M34.x, M35.1, M35.3, M36.0	Rheumatologic disease	1
K25.x-K28.x	Peptic ulcer disease	1
B18.x, K70.0-K70.3, K70.9, K71.3-K71.5, K71.7, K73.x, K74.x, K76.0, K76.2-K76.4, K76.8, K76.9, Z94.4	Mild liver disease	1
E10.0, E10.l, E10.6, E10.8, E10.9, E11.0, E11.1, E11.6, E11.8, E11.9, E12.0, E12.1, E12.6, E12.8, E12.9, E13.0, E13.1, E13.6, E13.8, E13.9, E14.0, E14.1, E14.6, E14.8, E14.9	Diabetes	1
E10.2-E10.5, E10.7, E11.2-E11.5, E11.7, E12.2-E12.5, E12.7, E13.2-E13.5, E13.7, E14.2-E14.5, E14.7	Diabetes with chronic complications	2
G04.1, G11.4, G80.1, G80.2, G81.x, G82.x, G83.0-G83.4, G83.9	Hemiplegia or paraplegia	2
I12.0, I13.1, N03.2-N03.7, N05.2-N05.7, N18.x, N19.x, N25.0, Z49.0-Z49.2, Z94.0, Z99.2	Renal disease	2
C00.x-C26.x, C30.x-C34.x, C37.x-C41.x, C43.x, C45.x-C58.x, C60.x-C76.x, C81.x-C85.x, C88.x, C90.x-C97.x	Any malignancy including leukemia and lymphoma	2
I85.0, I85.9, I86.4, I98.2, K70.4, K71.1, K72.1, K72.9, K76.5, K76.6, K76.7	Moderate or severe liver disease	3
C77.x-C80.x	Metastatic solid tumor	6
B20.x-B22.x, B24.x	Acquired Immunodeficiency syndrome (AIDS)	6

**Table A2:** Predictors of length of stay (days) for indication: bacteremic sepsis (univariate).

Predictors	Mean (95% CI)	*p*-Value
**BMI Group**		<0.001
Below 20	10.86 (10.53,11.20)	0.002
20–25	10.09 (9.71,10.46)	N/A
26–30	9.00 (8.60,9.40)	<0.001
31–35	7.96 (7.62,8.30)	<0.001
36–39	7.45 (7.06,7.84)	<0.001
40 and Above	8.55 (8.34,8.76)	<0.001
**Age Group, yrs**		<0.001
18–44 yrs	9.56 (9.18,9.94)	N/A
45–59 yrs	9.44 (9.19,9.70)	0.623
60–74 yrs	8.83 (8.62,9.04)	<0.001
75 yrs or older	8.25 (8.00,8.51)	<0.001
**Gender**		<0.001
Male	9.42 (9.22,9.62)	N/A
Female	8.56 (8.39,8.73)	<0.001
**Race**		<0.001
Non-white	9.51 (9.27,9.75)	N/A
White	8.64 (8.48,8.79)	<0.001
**Deyo-CCI**		<0.001
1	8.15 (7.84,8.46)	N/A
0	7.90 (7.54,8.25)	0.296
2 or higher	9.30 (9.15,9.46)	<0.001
***Comorbidities***		
**Atrial Fibrillation/Flutter**		<0.001
No	8.65 (8.50,8.79)	N/A
Yes	9.88 (9.61,10.15)	<0.001
**Chronic pulmonary disease**		0.052
No	9.00 (8.85,9.16)	N/A
Yes	8.72 (8.48,8.96)	0.052
**Congestive heart failure**		0.072
No	8.87 (8.73,9.01)	N/A
Yes	9.19 (8.87,9.50)	0.072
**Diabetes Mellitus**		<0.001
No	9.26 (9.10,9.42)	N/A
Yes	8.31 (8.09,8.53)	<0.001
**Hypertension**		<0.001
No	9.62 (9.46,9.79)	N/A
Yes	7.96 (7.76,8.16)	<0.001
**Peripheral vascular disorders**		0.016
No	8.87 (8.73,9.01)	N/A
Yes	9.41 (8.99,9.83)	0.016
**Prior MI**		<0.001
No	8.99 (8.86,9.12)	N/A
Yes	7.62 (7.05,8.19)	<0.001
**Renal failure**		<0.001
No	8.71 (8.56,8.86)	N/A
Yes	9.44 (9.20,9.69)	<0.001

CI = Confidence Interval; BMI = Body Mass Index; Deyo-CCI = Deyo Comorbidity Index; MI = Myocardial Infarction; N/A = Not Applicable.

**Table A3:** Predictors of length of stay for indication: bacteremic sepsis (multivariate).

Predictors	Mean (95% CI)	*p*-Value
**BMI Group**		<0.001
Below 20	9.73 (9.34,10.12)	0.004
20–25	8.98 (8.55,9.41)	N/A
26–30	7.91 (7.46,8.36)	<0.001
31–35	6.94 (6.55,7.33)	<0.001
36–39	6.32 (5.88,6.77)	<0.001
40 and Above	7.34 (7.05,7.63)	<0.001
**Age Group, yrs**		<0.001
18–44 yrs	8.56 (8.13,8.99)	N/A
45–59 yrs	8.38 (8.05,8.71)	0.458
60–74 yrs	7.81 (7.51,8.11)	0.001
75 yrs or older	6.73 (6.40,7.06)	<0.001
**Gender**		<0.001
Female	7.60 (7.34,7.86)	<0.001
Male	8.14 (7.86,8.43)	N/A
**Race**		0.076
Non-white	8.00 (7.69,8.32)	N/A
White	7.73 (7.50,7.97)	0.076
Deyo-CCI		<0.001
0	7.22 (6.82,7.62)	0.103
1	7.62 (7.27,7.98)	N/A
2 or higher	8.77 (8.52,9.01)	<0.001
***Comorbidities***		
**Atrial Fibrillation/Flutter**		<0.001
No	7.64 (7.40,7.88)	N/A
Yes	9.35 (8.98,9.71)	<0.001
**Congestive heart failure**		0.033
No	7.84 (7.61,8.08)	N/A
Yes	8.24 (7.83,8.66)	0.033
**Chronic pulmonary disease**		0.008
No	7.93 (7.69,8.18)	N/A
Yes	7.52 (7.18,7.87)	0.008
**Diabetes Mellitus**		<0.001
No	8.07 (7.83,8.32)	N/A
Yes	7.12 (6.78,7.45)	<0.001
**Hypertension**		<0.001
No	8.45 (8.18,8.72)	N/A
Yes	7.18 (6.90,7.46)	<0.001
**Prior MI**		<0.001
No	7.90 (7.67,8.14)	N/A
Yes	6.56 (5.93,7.18)	<0.001
**Peripheral vascular disorders**		0.154
No	7.85 (7.61,8.09)	N/A
Yes	8.18 (7.69,8.67)	0.154
**Renal failure**		<0.001
No	7.78 (7.54,8.02)	N/A
Yes	8.43 (8.06,8.79)	<0.001

CI = Confidence Interval; BMI = Body Mass Index; Deyo-CCI = Deyo Comorbidity Index; MI = Myocardial Infarction; N/A = Not applicable.
